# Mixed subtype thyroid cancer: A surveillance, epidemiology, and end results database analysis

**DOI:** 10.18632/oncotarget.21242

**Published:** 2017-09-23

**Authors:** Chunping Liu, Qiuyang Zhao, Zhi Li, Shuntao Wang, Yiquan Xiong, Zeming Liu, Tao Huang

**Affiliations:** ^1^ Department of Breast and Thyroid Surgery, Union Hospital, Tongji Medical College, Huazhong University of Science and Technology, Wuhan 430022, People’s Republic of China

**Keywords:** mixed subtype thyroid carcinoma, prognosis, SEER, PSM

## Abstract

The prognosis of patients with mixed subtype thyroid cancer (MSTC) is unclear. The present study compared the prognoses of MSTC, papillary thyroid cancer (PTC), and follicular thyroid cancer (FTC) to provide a new perspective regarding the treatment guidelines for these diseases. We evaluated data from patients with thyroid cancer who were included in the Surveillance, Epidemiology, and End Results database between 2004 and 2013. Patient mortality was evaluated using Cox proportional hazards regression analyses and Kaplan-Meier analyses with log-rank tests. The univariate Cox regression analysis showed that the cancer-specific survival rate for MSTC was lower than that for PTC and FTC. However, in the multivariate Cox regression analysis, the cancer-specific survival rate for MSTC was similar to that for PTC and FTC. Before matching influence factors, the cancer-specific survival rate for MSTC was lower than that for PTC and FTC. However, after propensity score matching for relevant factors, the cancer-specific survival rate for MSTC was also similar to that for PTC and FTC. Our result would be beneficial and provide a guideline for the understanding of MSTC.

## INTRODUCTION

Thyroid cancer is the most common endocrine malignancy, and its incidence has rapidly increased (more than 5% per year in both men and women). An estimated 64300 new thyroid cancer cases were diagnosed in the United States in 2016 according to Siegel et al. [1]. However, whether the increasing incidence of thyroid cancer is a result of the growing frequency of cancer detection procedures, such as high-resolution ultrasound, or is an actual increase in incidence is still controversial. Despite this uncertainty, the increasing rate of thyroid cancer is becoming a concerning issue.

Thyroid cancer has many histological subtypes; the most common of which are papillary thyroid cancer (PTC) and follicular thyroid cancer (FTC). Although many studies have focused on the morphological and clinicopathological risks as well as prognosis of PTC and FTC [2–9], mixed subtype thyroid cancer (MSTC), that is, adenocarcinoma combined with other types of carcinoma in the thyroid have rarely been investigated.

The Surveillance, Epidemiology, and End Results (SEER) Program has been established by the National Cancer Institute and is supported by the Surveillance Research Program to reduce the cancer burden, including that of thyroid cancer, among the US population. The SEER is a premier source of cancer surveillance data as well as analytical tools and is a leader in methodological expertise in collecting, analyzing, interpreting, and disseminating reliable population-based statistics.

In this study, we investigated the prognosis, that is, cancer-specific mortality and all-cause mortality, of MSTC compared with PTC and FTC using propensity score matching based on the SEER database. Our finding would provide a reference for further understanding and treatment of MSTC.

## RESULTS

### Demographic and clinical features

This study assessed data from 98888 patients with thyroid cancer to analyze the prognosis of MSTC. Of these patients, 92963 had PTC, 5865 had FTC, and 60 had MSTC. The mean age and follow-up duration according to different histological subtypes are shown in Table 1. No significant differences were noted in terms of the age between patients with MSTC and those with PTC or FTC.

**Table 1:** Characteristics for Patients with different histological types

Covariate	level	Histological types				
		MSTC(n=60)	PTC (n=92963)	P value	FTC (n=5865)	P value
Age (year)		53.66±18.13	49.36±15.29	0.085	51.33±17.20	0.397
Sex	Female	46(76.7%)	71785(77.2%)	0.919	4137(70.5%)	0.300
	Male	16(23.3%)	21178(22.8%)		1728(29.5%)	
Race	White	49(81.7%)	76038(82.9%)	0.033	4529(78.3%)	0.471
	Black	8(13.3%)	5704(6.2%)		695(12.0%)	
	Other	3(5.0%)	9987(10.9%)		558(9.7%)	
T stage	T1	17(28.8%)	55606(62.2%)	<0.001	1283(23.8%)	<0.001
	T2	12(20.3%)	13811(15.5%)		2174(40.2%)	
	T3	17(28.8%)	16407(18.4%)		1747(32.4%)	
	T4	13(22.1%)	3463(3.9%)		194(3.6%)	
N-stage	N0	42(72.4%)	69465(79.0%)	0.218	5370(97.0%)	<0.001
	N1	16(27.6%)	18460(21.0%)		168(3.0%)	
M-stage	M0	52(91.2%)	89658(98.8%)	<0.001	5377(94.2%)	0.334
	M1	5(8.8%)	1132(1.2%)		329(5.8%)	
Multifocality	No	28(50.0%)	52324(58.3%)	0.205	4735(85.6%)	<0.001
	Yes	28(50.0%)	37350(41.7%)		797(14.4%)	
Extension	No	37(62.7%)	75994(83.7%)	<0.001	5114(90.5%)	<0.001
	Yes	22(37.3%)	14847(16.3%)		539(9.5%)	
Radiation	None or refused	23(38.3%)	46761(51.5%)	0.006	2573(45.0%)	0.211
	Radiation Beam or Rdioactive implants	4(6.7%)	1705(1.9%)		177(3.1%)	
	Radioisotopes or Radiation beam plus isotopes or implants	33(55.0%)	42296(46.6%)		2970(51.9%)	
Surgery	Lobectomy	6(10.3%)	12688(14.2%)	<0.001	1300(23.6%)	0.037
	Subtotal or near-total thyroidectomy	2(3.4%)	3337(3.7%)		303(5.5%)	
	Total thyroidectomy	50(86.2%)	73162(82.1%)		3911(70.9%)	
Survival months (month)		41.63±37.44	49.09±33.76	0.041	53.12±34.33	0.006

MSTC: mixed subtype thyroid cancer; PTC: papillary thyroid cancer; FTC: follicular thyroid carcinoma.

### Cancer-specific and all-cause mortality rates of different histological subtypes

Upon follow-up on December 2013, the number of cancer-specific death was 7 in the MSTC group, 996 in the PTC group, and 190 in the FTC group. The cancer-specific mortality rates per 1000 person-years for MSTC, PTC, and FTC were 28.823 (95% confidence interval [CI]: 12.949–64.156), 2.403 (95% CI: 2.252–2.564), and 6.509 (95% CI: 5.598–7.569), respectively (Table 2). In addition, during the follow-up period, the number of patients who died from all-cause death in the MSTC, PTC, and FTC group was 11, 4388, and 538, respectively. The all-cause mortality rates per 1000 person-years for MSTC, PTC, and FTC were 48.038 (95% CI: 25.8547–89.281), 11.068 (95% CI: 10.739–11.408), and 19.337 (95% CI: 17.717–21.104), respectively (Table 2).

**Table 2:** Hazard Ratios of different histological types for the cancer specific deaths and all cause deaths of thyroid cancer

Histological types	Cancer-Specific Deaths,	%	Cancer-Specific Deaths per	95% CI	All Cause Deaths,	%	All Cause Deaths per	95% CI
	No.		1,000 Person-Years		No.		1,000 Person-Years	
MSTC	7	11.7	28.823	12.949-64.156	11	18.33	48.038	25.847-89.281
PTC	966	1.04	2.403	2.252-2.564	4388	4.72	11.068	10.739-11.408
FTC	190	3.24	6.509	5.598-7.569	538	9.17	19.337	17.717-21.104

MSTC: mixed subtypes thyroid cancer; PTC: papillary thyroid cancer; FTC: follicular thyroid carcinoma.

### Risk factors for cancer-specific and all-cause mortality

The results of the univariate Cox regression analyses demonstrated that cancer-specific mortality was associated with age; sex; race; histological type; tumor, node, and metastases (TNM) stage; extension; radiation treatment; and surgical approach. Moreover, all-cause mortality was also found to be associated with age, sex, race, histological type, TNM stage, multifocality, extension, radiation treatment, and surgical approach. Meanwhile, multivariate Cox regression model showed that histological subtypes were not an independent risk factor for cancer-specific and all-cause mortality (Table 3).

**Table 3:** Risk factors for survival: outcome of thyroid cancer specific mortality and all-cause mortality

Covariate	Level	Thyroid Cancer specific mortality						All cause mortality	
		Univariate Cox regression		Multivariate Cox regression		Univariate Cox regression		Multivariate Cox regression	
		Hazard Ratio (95% CI)	p-value	Hazard Ratio (95% CI)	p-value	Hazard Ratio (95% CI)	p-value	Hazard Ratio (95% CI)	p-value
Age		1.097(1.092-1.102)	<0.001	1.067(1.061-1.073)	<0.001	1.087(1.085-1.089)	<0.001	1.077(1.074-1.080)	<0.001
Sex	Female	ref		ref		ref		ref	
	Male	2.765(2.463-3.103)	<0.001	1.407(1.201-1.647)	<0.001	2.466(2.330-2.609)	<0.001	1.662(1.549-1.783)	<0.001
Race	White	ref		ref		ref		ref	
	Black	1.077(0.853-1.359)	0.533	1.129(0.803-1.586)	0.486	1.295(1.170-1.435)	<0.001	1.417(1.248-1.608)	<0.001
	Other	1.472(1.249-1.733)	<0.001	0.896(0.712-1.129)	0.353	0.958(0.872-1.053)	0.375	0.791(0.698-0.896)	<0.001
histological types	MSTC	ref		ref		ref		ref	
	PTC	0.077(0.037-0.163)	<0.001	0.474(0.151-1.489)	0.201	0.221(0.123-0.400)	<0.001	0.494(0.221-1.105)	0.086
	FTC	0.229(0.108-0.487)	<0.001	0.866(0.271-2.767)	0.808	0.400(0.220-0.726)	0.003	0.623(0.0.277-1.401)	0.252
T-stage T-stage	T1	ref		ref		ref		ref	
	T2	2.881(2.164-3.836)	<0.001	2.145(1.524-3.020)	<0.001	1.087(0.992-1.191)	0.075	1.093(0.982-1.217)	0.102
	T3	7.763(6.173-9.763)	<0.001	3.574(2.494-5.122)	<0.001	1.580(1.461-1.709)	<0.001	1.168(1.015-1.343)	0.030
	T4	86.012(69.700-106.142)	<0.001	13.459(8.952-20.234)	<0.001	7.577(6.999-8.203)	<0.001	2.687(2.233-3.233)	<0.001
N stage	N0	ref		ref		ref		ref	
	N1	4.661(4.104-5.293)	<0.001	1.992(1.658-2.393)	<0.001	1.662(1.555-1.777)	<0.001	1.497(1.364-1.644)	<0.001
M-stage	M0	ref		ref		ref		ref	
	M1	50.222(44.405-56.800)	<0.001	6.865(5.691-8.281)	<0.001	13.519(12.422-14.713)	<0.001	3.926(3.435-4.488)	<0.001
Multifocality	No	ref		ref		ref		ref	
	Yes	0.893(0.783-1.019)	0.092	0.781(0.666-0.915)	0.002	0.883(0.830-0.939)	<0.001	0.968(0.901-1.041)	0.38
Extension	No	ref		ref		ref		ref	
	Yes	12.895(11.270-14.755)	<0.001	1.574(1.137-2.178)	0.006	2.603(2.446-2.770)	<0.001	1.121(0.958-1.310)	0.153
Radiation	None or refused	ref		ref		ref		ref	
	Radiation Beam or Rdioactive implants	14.149(12.142-16.486)	<0.001	2.964(2.335-3.763)	<0.001	3.460(3.109-3.850)	<0.001	1.496(1.280-1.747)	<0.001
	Radioisotopes or Radiation beam+ isotopes/implants	0.902(0.790-1.030)	0.126	0.825(0.682-0.996)	0.046	0.584(0.550-0.621)	<0.001	0.696(0.644-0.753)	<0.001
Surgery	Lobectomy	ref		ref		ref		ref	
	Subtotal or near-total thyroidectomy	1.896(1.355-2.653)	<0.001	1.190(0.786-1.801)	0.412	1.023(0.880-1.190)	0.764	1.021(0.862-1.209)	0.812
	Total thyroidectomy	1.385(1.118-1.717)	0.003	1.041(0.793-1.367)	0.772	0.820(0.755-0.889)	<0.001	0.967(0.879-1.064)	0.493

MSTC: mixed subtypes thyroid cancer; PTC: papillary thyroid cancer; FTC: follicular thyroid carcinoma;

### Adjusting for patient characteristics using propensity score matching

**Figure 1:**
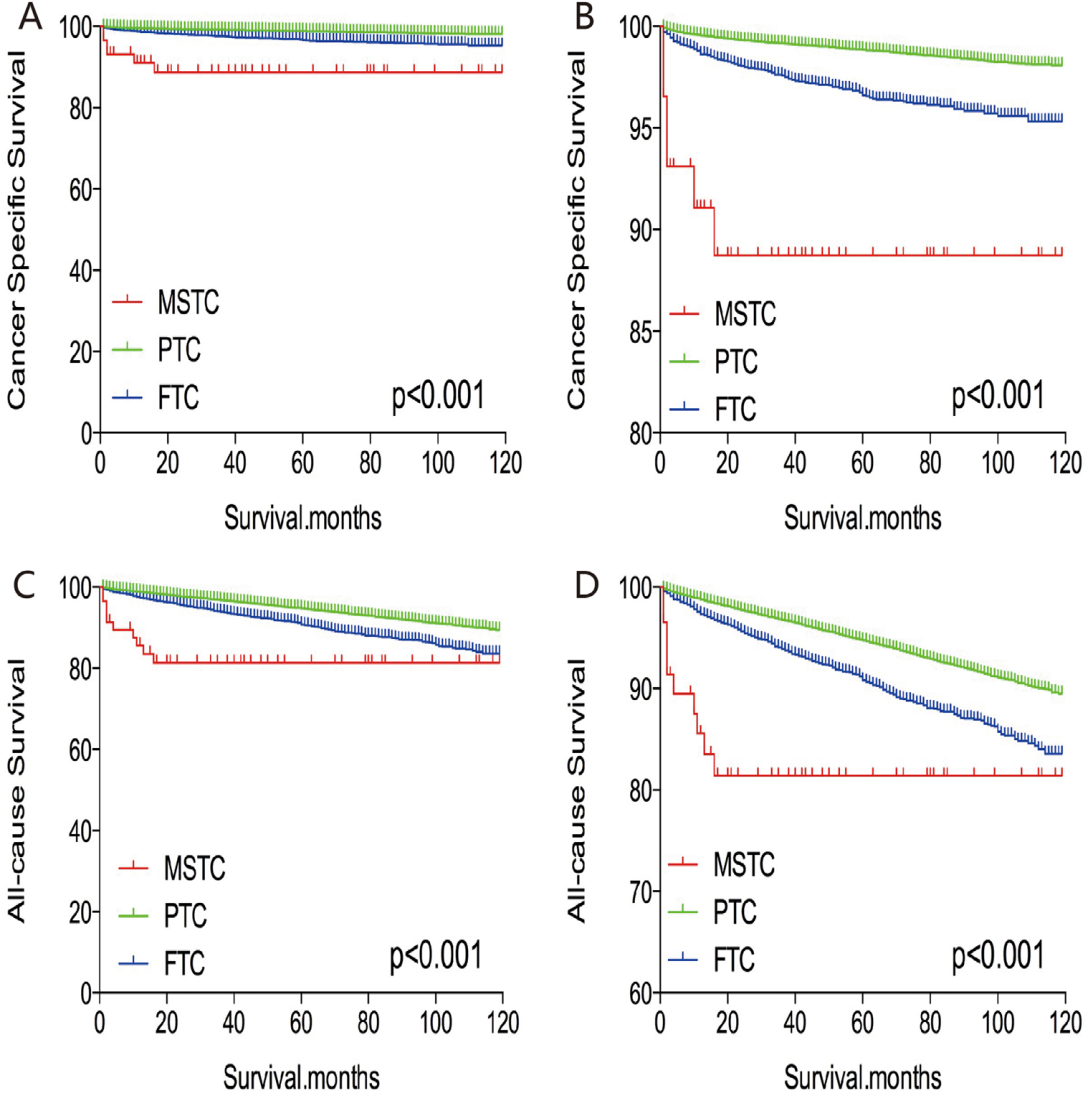
Kaplan Meier curves among patients stratified by subtype for cancer-specific mortality **(A, B)** and all-cause mortality **(C, D)**.

**Figure 2:**
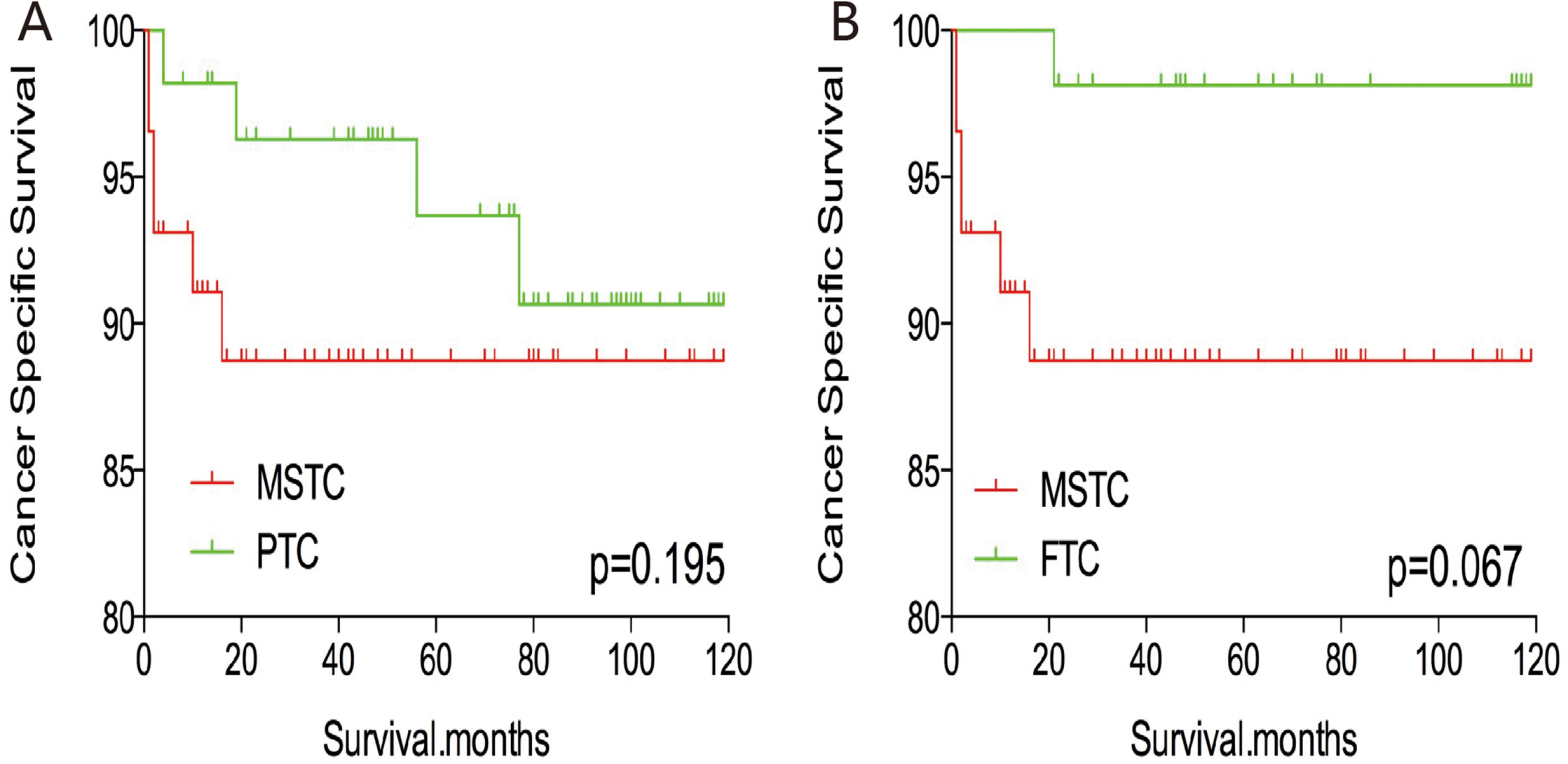
Kaplan Meier curves of cancer-specific mortality for matched subtype pairs Age, sex and race matching between MSTC and PTC **(A)**, MSTC and FTC **(B)**.

**Figure 3:**
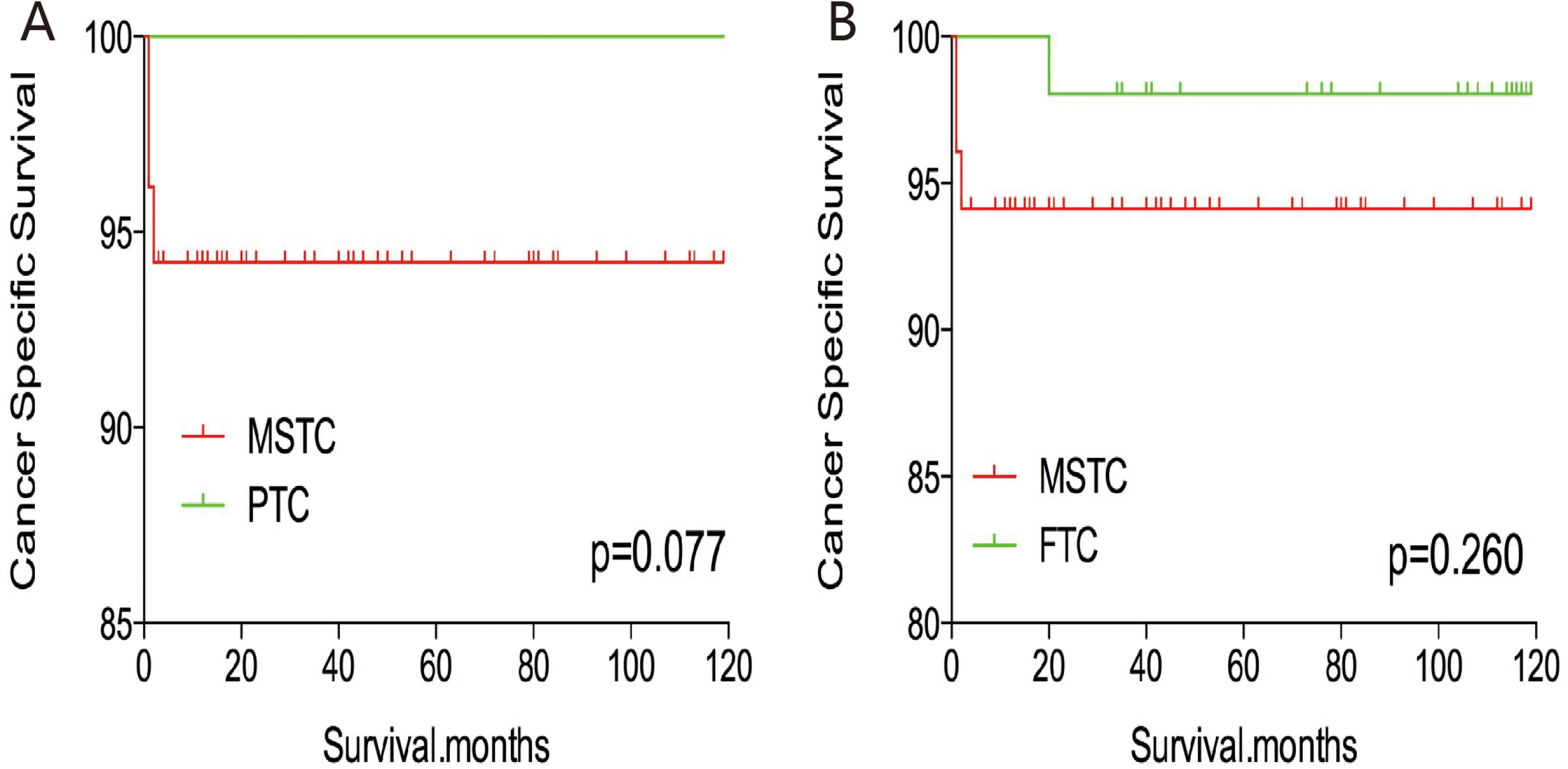
Kaplan Meier curves of cancer-specific mortality for matched subtype pairs Age, sex, race, T/N/M stage, multifocality, extension matched between MSTC and PTC **(A)**, MSTC and FTC **(B)**.

**Figure 4:**
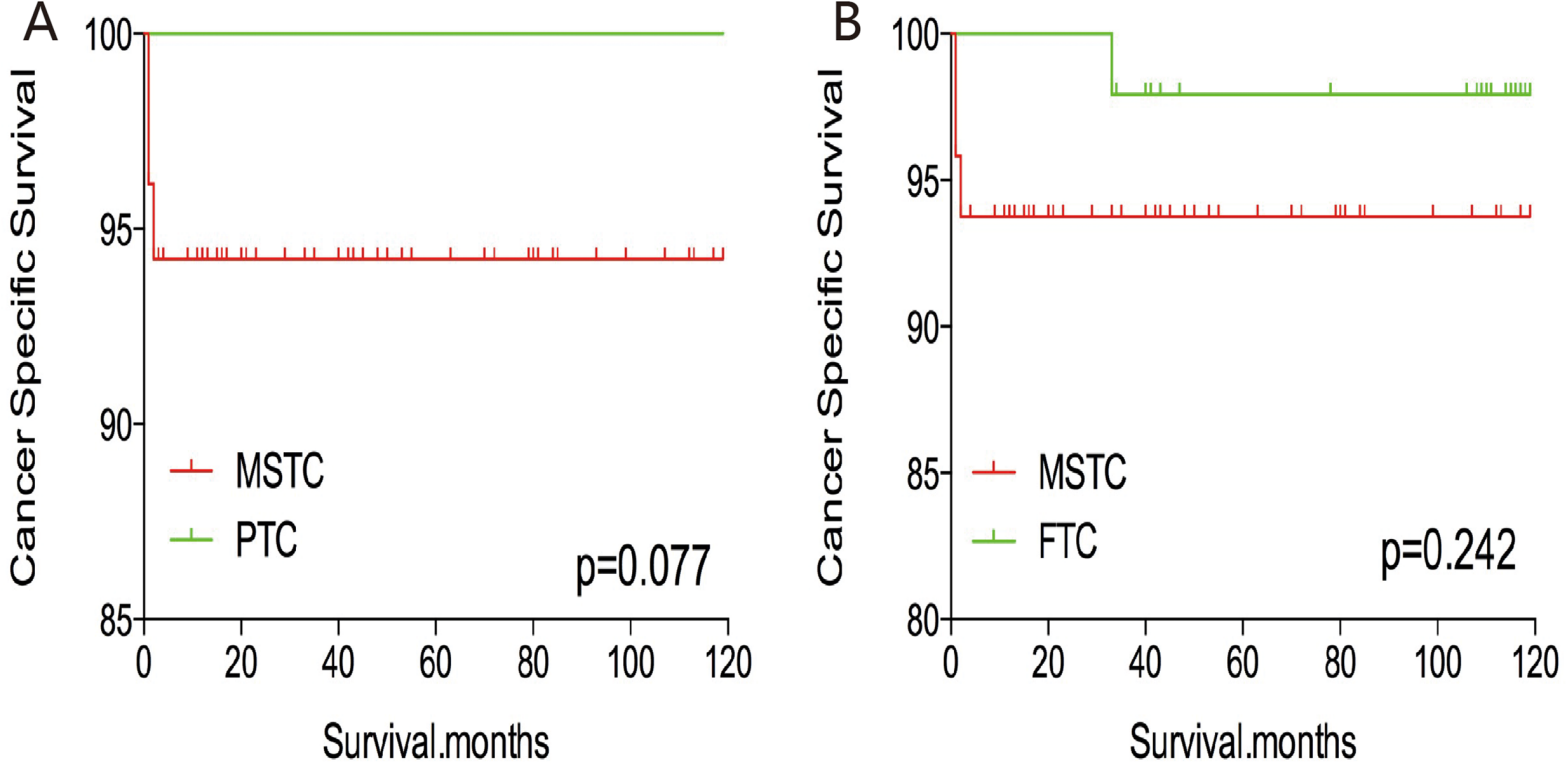
Kaplan Meier curves of cancer-specific mortality for matched Subtype pairs Age, sex, race, T/N/M stage, multifocality, extension, surgery and radiation treatment matched between MSTC and PTC **(A)**, MSTC and FTC **(B)**.

Without matching any factors, the cancer-specific and all-cause mortality rates between that of MSTC and that of PTC and FTC were significantly different (both p < 0.001, Figure 1A–1D). Thus, to minimize selection bias, propensity score matching (PSM) was performed for age, sex, race, TNM stage, multifocality, extension, and radiation treatment. After PSM for demographic data, such as age, sex, and race, the cancer-specific mortality rate between MSTC and PTC and FTC decreased (p=0.195 and 0.067, respectively; Figure 2A, 2B). After PSM for age, sex, race, and clinicopathological features (TNM stage, multifocality, and extension), the cancer-specific mortality rate between MSTC and PTC and FTC was still not significantly different (p=0.077 and p=0.260, respectively; Figure 3A, 3B). After PSM for all relevant factors including radiation and surgery treatment, the cancer-specific mortality rate for MSTC remained not significantly different compared with PTC (p=0.077) and FTC (p=0.242) (Figure 4A, 4B).

**Figure 5:**
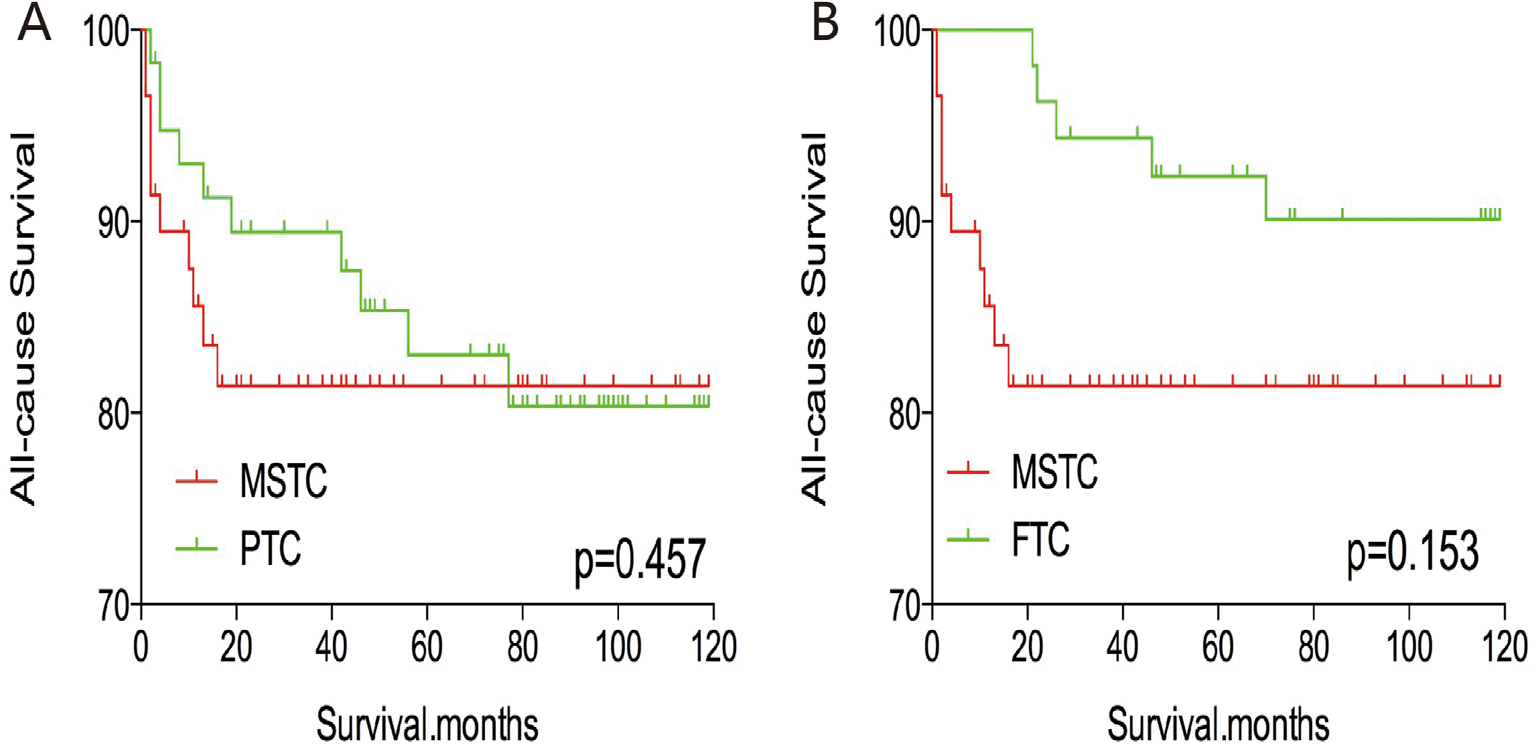
Kaplan Meier curves of all-cause mortality for matched Subtype pairs Age, sex and race matching between MSTC and PTC **(A)**, MSTC and FTC **(B)**.

**Figure 6:**
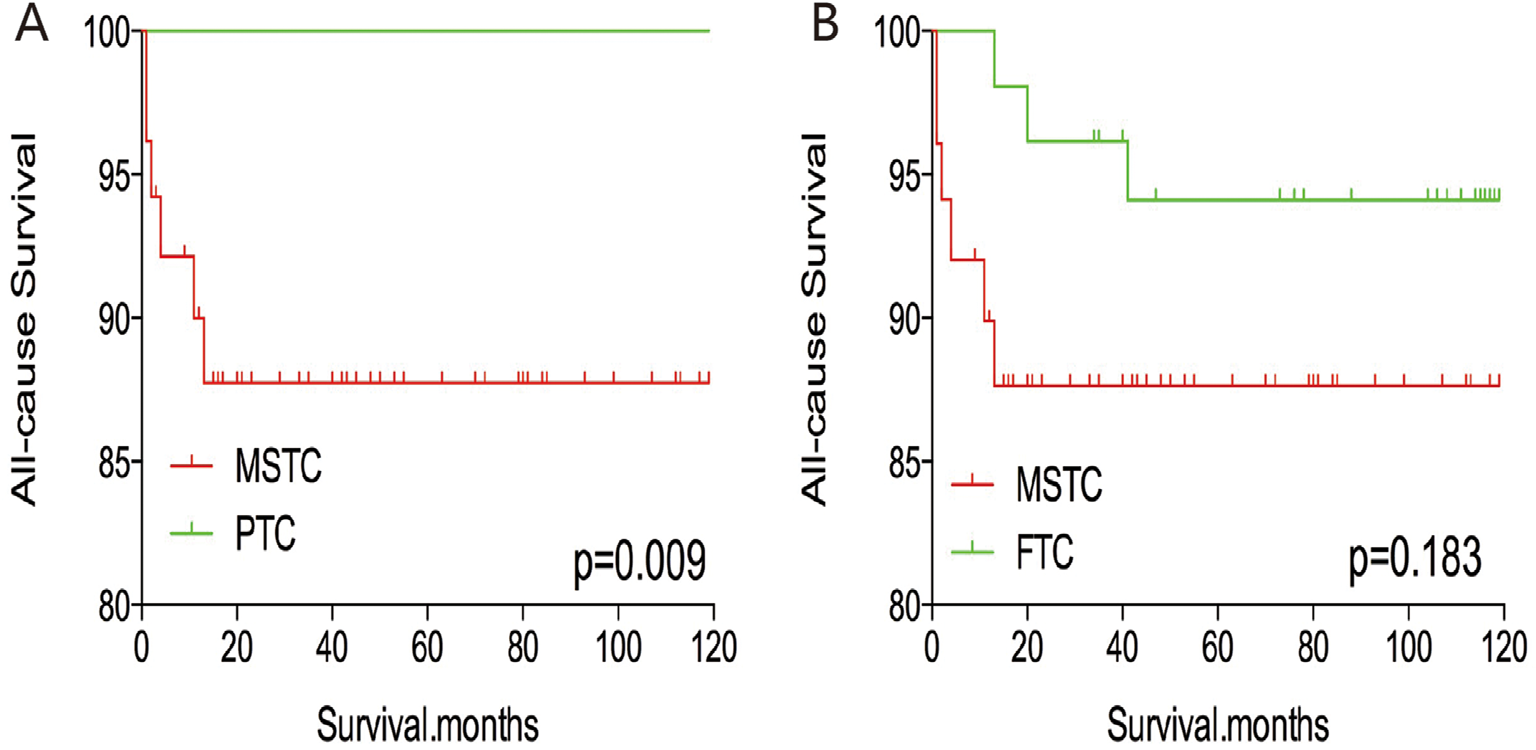
Kaplan Meier curves of all-cause mortality for matched Subtype pairs Age, sex, race, T/N/M stage, multifocality, extension matching between MSTC and PTC **(A)**, MSTC and FTC **(B)**.

**Figure 7:**
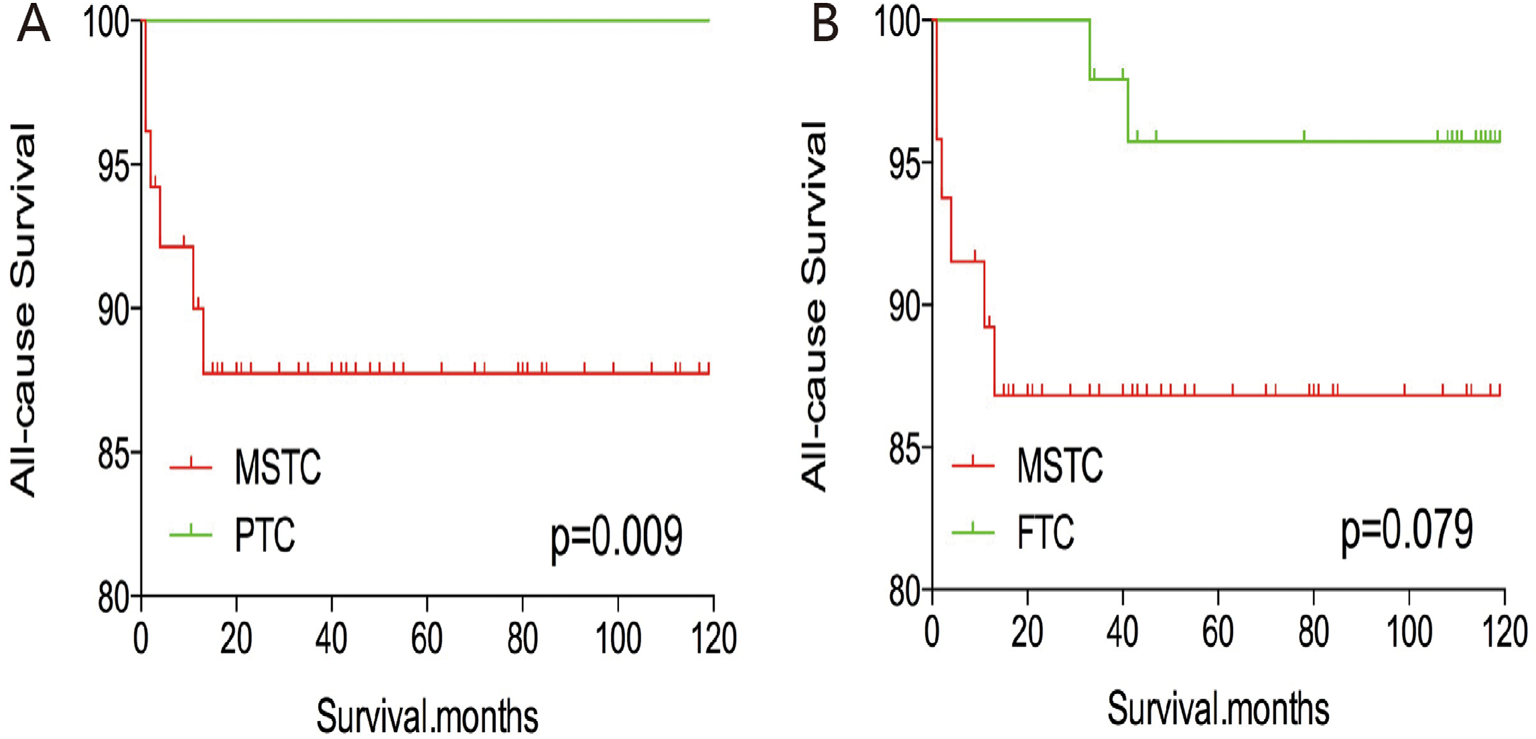
Kaplan Meier curves of all-cause mortality for matched Subtype pairs Age, sex, race, T/N/M stage, multifocality, extension, surgery and radiation treatment matching between MSTC and PTC **(A)**, MSTC and FTC **(B)**.

After PSM for demographic data (age, sex, and race), the all-cause mortality rate for MSTC was similar to that of PTC and FTC (p=0.457 and 0.153, respectively; Figure 5A, 5B). However, after PSM for age, sex, race, and clinicopathological factors (TNM stage, multifocality, and extension) (Figure 6A, 6B), MSTC showed a lower all-cause survival than PTC (p=0.009) and a similar all-cause survival compared with FTC (p=0.183). similar results were obtained after PSM for all relevant factors including radiation and surgery treatment: MSTC was associated with a lower all-cause survival rate compared with PTC (p=0.009) and a similar all-cause survival when compared with FTC (p=0.079) (Figure 7A, 7B).

## DISCUSSION

The histogenetic and pathogenetic origin of MSTC has been a topic of interest and has remained controversial. Volante et al. suggested “the hostage hypothesis” that stipulates that MSTC originate from different cells [10]. In their study, they suggested that MSTC are not derived from a single stem cell because the clonality analysis shows that different subtypes exhibit different patterns of molecular mutations and X-chromosomal inactivation.

In addition, whether MSTC has unique biologic behaviors compared with other subtypes is still unclear. Currently, only few investigations focus on the prognosis of MSTC [10-12]. Furthermore, the discussion of the American Thyroid Committee guideline on the biological behavior, molecular features, and prognosis of MSTC is limited.

In our study, analysis results of information obtained from the SEER database showed that histological subtype was not an independent factor for cancer-specific and all-cause mortality rates of MSTC. Before matching risk factors, MSTC had worse cancer-specific and all-cause mortality compared with PTC and FTC. However, after matching for all influencing factors, including radiation treatment, MSTC had similar prognosis to PTC and FTC. These results indicate that if patients with MSTC had similar demographic features, clinicopathological factors, and radiation treatment with those of patients with PTC and FTC, the survival rate would not be significantly different. Such results would be helpful for clinicians during the decision-making process.

One dilemma of MSTC is the diagnosis by fine-needle aspiration cytology. For example, mixed medullary-follicular thyroid carcinoma characterized by coexistence of morphological features of both follicular thyroid cancer and medullary thyroid cancer. It is difficult to identify without immunocytochemical examinations. When fine-needle aspiration cytology is preferred to diagnose such malignancy, adequate specimen and determination of serum calcitonin and thyroglobulin levels would be help for establishing an accurate diagnosis [13-17].

Notably, whether the type of treatment, including surgical approaches and radiation, affects the prognosis of MSTC still lacks adequate evidence [10-12, 17-20]. In our study, radiation was more frequently performed in patients with MSTC (61.7%) than in patients with PTC (48.5%). Moreover, MSTC patients (86.2%) were more likely to undergo total thyroidectomy than patients with PTC (82.1%) and FTC (70.9%). Therefore, the similar cancer-specific mortality of MSTC to PTC and FTC after matching for treatment approaches may suggest that patients with MSTC require aggressive treatment. It means that the difference of cancer-specific mortality between MSTC and PTC, FTC was shrink down may due to the aggressive treatment of MSTC.

Our study has some limitations. First, the lack of information on recurrence may cause overestimation bias when evaluating cancer-specific and all-cause mortality. Furthermore, we cannot differentiate the specific subtypes. Clonality analysis of mixed type thyroid cancer was also not performed; thus, analysis for each type compared with PTC and FTC separately could not be performed. In addition, BRAF mutation, TERT promoter mutation, and other molecular markers were not investigated in our study or matched for analysis.

In conclusion, we have shown the prognosis of MSTC compared with PTC and FTC. Our result would be beneficial for developing a personalize and accurate treatment guideline and understanding of MSTC.

## MATERIALS AND METHODS

### Ethical considerations, study population and data collection

This study’s retrospective protocol was approved by Union hospital’s ethical review board and complied with the ethical standards of the Declaration of Helsinki, as well as the relevant national and international guidelines.

The present study evaluated SEER data (2004–2013) from patients with thyroid cancer according to their subtype (MSTC, PTC, and FTC) using code C73.9 from the International Classification of Diseases for Oncology(i.e., thyroid, papillary, and/or follicular histology). The eligible diagnostic codes were: “papillary carcinoma”, “papillary adenocarcinoma”, “follicular adenocarcinoma”, “papillary carcinoma, follicular variant”, and “papillary & follicular adenocarcinoma” “adenocarcinoma with mixed subtypes”. Cases without American Joint Committee on Cancer staging information (version 6) were excluded to ensure accurate analyses. Cases without information of follow up time were also excluded. The three histological subtypes were compared according to age, sex, race, TNM stage, multifocality, extension, and radiation treatment (i.e., none or refused, external beam radiation therapy, or RAI).

### Statistical analyses

The quantitative variables were expressed as mean ± standard deviation (SD), while the categorical ones were presented as percentages. Patient survival curves for thyroid cancer-specific mortality and all-cause mortality were examined by Kaplan-Meier analyses with the log-rank test. Cox proportional hazard regression analyses were using to estimate hazard ratios and 95% CIs, in order to quantify the effects of the different histological subtypes on cancer-specific and all-cause mortality. PSM was also used to further adjust for potential baseline confounding factors. All p-values were 2-sided, and p-values < .05 were considered significant. Analyses were performed using SPSS version 23.0, Stata/SE version 12 (Stata Corp.), and GraphPad Prism version 6 (GraphPad Software Inc.).

## Abbreviations

MSTC: Mixed subtype thyroid cancer
PTC: Papillary thyroid cancer
FTC: Follicular thyroid cancer (FTC)
SEER: Surveillance, Epidemiology, and End Results
CI: Confidence interval
TNM: tumor, node, and metastases
PSM: Propensity score matching
