# Correction: Mixed subtype thyroid cancer: A surveillance, epidemiology, and end results database analysis

**DOI:** 10.18632/oncotarget.25001

**Published:** 2018-03-23

**Authors:** Chunping Liu, Qiuyang Zhao, Zhi Li, Shuntao Wang, Yiquan Xiong, Zeming Liu, Tao Huang

**Affiliations:** ^1^ Department of Breast and Thyroid Surgery, Union Hospital, Tongji Medical College, Huazhong University of Science and Technology, Wuhan 430022, China

**This article has been corrected:** The proper name of the institution is as follows:

^1^ Department of Breast and Thyroid Surgery, Union Hospital, Tongji Medical College, Huazhong University of Science and Technology, Wuhan 430022, China

Original article: Oncotarget. 2017; 8:86556-86565. https://doi.org/10.18632/oncotarget.21242

